# Prospective associations of adiposity with health-related quality of life during breast cancer survivorship among Black women

**DOI:** 10.1007/s11764-025-01829-x

**Published:** 2025-08-06

**Authors:** Carola T. Sánchez-Díaz, Bo Qin, Nur Zeinomar, Hari S. Iyer, Marley Perlstein, Karen S. Pawlish, Kitaw Demissie, Chi-Chen Hong, Elisa V. Bandera

**Affiliations:** 1 Section of Cancer Epidemiology and Health Outcomes, Rutgers Cancer Institute, 120 Albany St, New Brunswick, NJ 08901 USA; 2 Department of Medicine, Robert Wood Johnson Medical School, Rutgers University, New Brunswick, NJ USA; 3 Department of Health, New Jersey State Cancer Registry, New Jersey , Trenton, NJ USA; 4 Department of Epidemiology and Biostatistics, SUNY Downstate Health Sciences University School of Public Health, Brooklyn, NY USA; 5 Department of Cancer Prevention and Control, Roswell Park Comprehensive Cancer Center, Buffalo, NY USA; 6 Division of Cancer Control and Population Sciences, University of Puerto Rico Comprehensive Cancer Center, San Juan, Puerto Rico

**Keywords:** Breast cancer survivors, Black women, Health-related quality of life, Adiposity, Obesity, Body composition

## Abstract

**Background:**

Black breast cancer (BC) survivors face a disproportionately high burden of obesity, which may detrimentally impact health-related quality of life (QoL). This study examines the relationship between various adiposity measures—body mass index (BMI), waist circumference, waist-to-hip ratio (WHR), percentage body fat (BF), and fat mass index (FMI)—and QoL in Black BC survivors participating in the Women’s Circle of Health Follow-Up Study, a New Jersey-based population cohort.

**Methods:**

We analyzed data from 785 women aged 20–75, diagnosed with BC between 2012 and 2019. Participants completed interviews approximately 10 months (visit 1) and 24 months (visit 2) post-diagnosis. Adiposity measures were recorded at visit 1, while QoL was evaluated at visit 2 using the Functional Assessment of Cancer Therapy–Breast (FACT-B) overall score and subscales (physical, emotional, functional, social well-being, and BC-specific subscale). Multivariable robust Poisson regression models were used to estimate risk ratios for the association between adiposity and QoL, dichotomized as low/high based on sample medians.

**Results:**

High levels of general and central obesity were prevalent (58% and 75%, respectively). Women with BMI ≥ 40 kg/m^2^ had a 54% higher risk of lower overall QoL compared to those with BMI 18.5–24.9 (RR, 1.54; 95% CI, 1.19–2.00). Elevated risk of low QoL was also observed for higher quartiles of waist circumference, BF, and FMI, primarily in physical well-being and BC-specific subscales.

**Conclusion:**

Higher adiposity is significantly associated with lower QoL among Black BC survivors.

**Implications for Cancer Survivors:**

Maintaining a healthy weight may support long-term QoL in Black BC survivors.

## Background

With the increasing number of breast cancer (BC) survivors in the United States (US) [1], understanding underlying factors affecting BC survivorship, including excess adiposity and health-related quality of life (QoL), has become a public health priority. Minoritized populations in the US suffer from a disproportionate burden of obesity [2], which may impact QoL [3], but there is limited research on Black BC survivors. Obesity is a well-established modifiable risk factor associated with incident postmenopausal BC and cancer-associated death and is known to pose challenges for the clinical management of BC and prognosis [4–7]. Minoritized populations, particularly Black women, are also more likely to have general and central obesity and to experience worse BC survival than any other racial or ethnic group [2, 8–10].

BC survivors often experience symptoms such as pain and psychosocial challenges related to their diagnosis and treatments [11, 12], which may be exacerbated by excess adiposity [13]. Black BC survivors with obesity, in particular, are more likely to have obesity-related comorbidities such as hypertension and diabetes compared to non-obese women [9]. In addition, patient-reported outcomes such as QoL, physical functioning, cancer-related symptoms, and other psychosocial factors are increasingly recognized as essential components of cancer survivorship [11]. This underscores the critical need to address modifiable lifestyle factors, particularly obesity, to improve QoL and the psychosocial well-being among Black BC survivors.

There is an increasing focus in the clinical setting on improving QoL and the overall holistic well-being of BC survivors [14], promoting a more comprehensive, patient-centered approach to post-treatment care. Despite this growing interest, the evidence regarding the role of adiposity in QoL among Black BC survivors remains inconclusive and continues to evolve. Compared to White women and other racial and ethnic groups, Black women are consistently more likely to be obese and more likely to report poor QoL [3, 11, 15]. Consequently, understanding obesity as an important prognostic factor for BC outcomes in Black women remains an area of active research that warrants further investigation.

This study aims to comprehensively examine how adiposity influences QoL among Black BC survivors. Given that body mass index (BMI) has been shown to be an imprecise measure of adiposity, failing to account for variations in body fat distribution and composition [16], this is particularly relevant in our cohort, where nearly half of participants reported distress over weight changes since diagnosis. We evaluated prospective associations between BMI, body fat distribution (waist circumference and waist-to-hip ratio), and body composition (percent body fat and fat mass index) with QoL in the Women’s Circle of Health Follow-Up Study, a population-based cohort of Black BC survivors in New Jersey.

## Methods

### Study population

This prospective study enrolled participants from the Women’s Circle of Health Follow-up Study (WCHFS), a population-based cohort of Black BC survivors in New Jersey. Detailed study methods have been previously described [17]. In brief, participants were identified through rapid case ascertainment in 10 counties of New Jersey by the New Jersey State Cancer Registry (NJSCR). Eligible participants were women who self-identified as Black or African American, were diagnosed with histologically confirmed ductal carcinoma in situ or invasive BC, aged 20–75 years at diagnosis, able to speak English, and had no prior history of cancer except for nonmelanoma skin cancer.

This analysis used data from the baseline and a follow up visit at approximately 10 and 24 months since diagnosis, respectively. These visits included collection of anthropometric measures, blood pressure readings, and biospecimen collection following standardized protocols [17]. Additionally, computer-assisted questionnaires were administered to collect data on sociodemographic characteristics, reproductive history, lifestyle factors (e.g., alcohol and smoking), patient-reported outcomes (e.g., QoL), and medical history. Anxiety and depression were measured using the Hospital Anxiety and Depression Scale (HADs) [18–20]. In this prospective analysis, we used adiposity measures from baseline visit and QoL from follow up visit.

Clinicopathological information was obtained from pathology records or the NJSCR and included American Joint Committee on Cancer (AJCC) stage, tumor grade, and the status of estrogen receptor, progesterone receptor, and human epidermal growth receptor 2 (HER-2) for tumor subtype. Data on BC treatment was collected from medical records or self-reported if missing, with a prior study by our team showing good concordance between self-reported treatment and medical records [8].

All participants provided informed consent, and the study protocol was approved by the Institutional Review Boards of Rutgers University and Roswell Park Comprehensive Cancer Center.

A total of 794 WCHFS participants diagnosed between May 2012 and May 2019 were included in the study. Participants with missing QoL measures (*n* = 9) were excluded, resulting in an analytical sample of 785 participants.

### Anthropometric measures

In this analysis, we use anthropometric measures taken at baseline home visits, at approximately 10 months post-diagnosis, by trained interviewers following a standardized protocol [17]. These measurements included weight, height, body fat distribution (waist and hip circumferences), and body composition (fat and lean mass, percent body fat), assessed using a portable bioelectrical impedance analysis scale (TBF-300 A Total Body Composition Analyzer; Tanita). For missing BMI values, self-reported weight and height from the home interview were used to calculate values, as prior analyses showed high concordance between BMI derived from self-reported and measured values (intraclass correlation: 0.97) [21]. Waist and hip circumferences were measured twice, and if the difference between the two measurements was greater than 2 cm, a third measurement was taken. The average of the three measurements was then used for the analysis.

### Health-related quality of life

QoL was assessed at the follow-up 1 interview, approximately 24 months post-diagnosis, using the Functional Assessment of Cancer Therapy-Breast Cancer (FACT-B) instrument. This tool measures five domains: physical well-being (PWB), social and family well-being (SFWB), emotional well-being (EWB), functional well-being (FWB), and the BC -subscale (BCS)] [22–24]. The BCS subscale captures the unique physical and psychosocial impacts of BC and its treatment that are not addressed by general QoL instruments, including items related to body image (e.g., changes in appearance, surgical scars, breast loss), arm swelling or sensitivity following treatment, the impact of cancer on family and personal relationships, emotional concerns such as fear of recurrence, and changes in sexuality or femininity post-treatment. Increased FACT-B and subscale scores indicate better QoL outcomes.

### Statistical analysis

ANOVA was used to assess the relationships between sociodemographic characteristics and FACT-B total and domain scores. FACT-B scores were dichotomized into high and low categories based on the sample median. BMI was calculated as weight in kilograms divided by height in meters squared (kg/m^2^) and classified according to the World Health Organization definitions: normal weight (BMI, 18.5–24.9), overweight (BMI, 25.0–29.9), obese class I (BMI, 30.0–34.9), obese class II (BMI, 35.0–39.9), and obese class III (≥ 40.0) [25, 26]. Waist-to-hip ratio was calculated as waist circumference divided by hip circumference, and fat mass index was computed as fat mass in kilograms divided by height in meters squared. Body composition (percent body fat and fat mass index) and body fat distribution (waist-to-hip ratio and waist circumference) were evaluated in quartiles and analyzed using an ordinal test for trend.

Multivariable Poisson regression models with robust standard errors were used to estimate risk ratios and 95% confidence intervals for the association between anthropometric measures and QoL.

We selected a priori the following covariates to be considered: age at diagnosis (continuous), educational level, household income, insurance status, menopausal status, foreign-born status, marital status, lifestyle factors relevant to QoL (smoking status: former, current, never), alcohol consumption in the past year, physical activity (METS—hours/week), physical activity (guideline adherence), comorbidities associated with obesity (history of hypertension and diabetes), lymphedema since diagnosis, stage at diagnosis, tumor grade, tumor subtype (Luminal A, HER2-enriched, triple negative), and cancer treatment information (type of surgery, chemotherapy, radiation, and endocrine therapy).

A reduced set of covariates was selected based on backward elimination (*p*-value < 0.1). All final models were adjusted for age, education, marital status, menopausal status, household income, smoking status, tumor stage, subtype, surgery, and chemotherapy. In addition, we adjusted for diabetes and hypertension to account for their role in the association of obesity with QoL. We conducted a sensitivity analysis by excluding women with abnormal anxiety or depression scores to assess how mental health conditions might influence the association between adiposity and QoL.

Additionally, the associations between obesity and QoL were stratified by stage at diagnosis, with multiplicative interaction tested using likelihood ratio tests. We set α = 0.05 to evaluate the statistical significance for all hypothesis tests. All statistical analyses were performed using Stata, version 18.0.

## Results

Among the 785 Black BC survivors in our study, the mean age was 55.5 years (SD = 10.7), with a mean FACT-B total score of 113.2 (SD = 23.1), reflecting the participant’s overall QoL. The mean BMI was 32.0 kg/m^2^ (SD = 6.7), indicating a high prevalence of obesity. Specifically, 58.3% of women were obese (BMI > 30 kg/m^2^) and 75% of participants had a WHR greater than 0.85 (Table 1), signifying a high prevalence of central obesity.

**Table 1:** Associations of demographic, lifestyle and clinical characteristics with FACT-B Quality of Life total score and domains among participants in the Women’s Circle of Health Follow-Up Study

	Overall	Overall FACT-B		Physical well-being		Functional well-being		Social well-being		Emotional well-being		Breast cancer specific	
	*n* = 785	(score 0 = 148)		(score 0–28)		(score 0–28)		(score 0–28)		(score 0–24)		(score 0–40)	
	*n*	Mean (SE)	*p* value	Mean (SE)	*p* value	Mean (SE)	*p* value	Mean (SE)	*p* value	Mean (SE)	*p* value	Mean (SE)	*p* value
Overall score		113.2 (23.1)		21.8 (5.6)		20.7 (6.2)		22.6 (5.4)		20.0 (4.2)		27.6 (7.4)	
Age at diagnosis (years)			< 0.0001		0.002		0.09		0.58		< 0.0001		< 0.0001
21–40	66	105.5 (25.7)		20.4 (6.1)		20.7 (6.8)		22.2 (5.9)		17.8 (5.1)		24.3 (7.1)	
41–60	436	110.8 (23.9)		21.5 (6.0)		20.3 (6.5)		22.6 (5.5)		19.5 (4.3)		26.9 (7.7)	
61–75	283	117.8(19.6)		22.7 (4.8)		21.4 (5.7)		22.8 (5.2)		21.2 (3.3)		29.4 (6.4)	
**Education**			< 0.001		< 0.001		< 0.001		0.001		0.001		< 0.0001
≤ High school graduate	276	110.8 (23.9)		21.6 (5.6)		20.3 (6.3)		21.9 (5.9)		19.6 (4.6)		27.3 (7.5)	
Some college	266	109.2 (24.2)		20.8 (6.3)		20.0 (6.6)		22.5 (5.4)		19.6 (4.2		26.3 (7.6)	
≥ College	242	119.4 (19.1)		23.2 (4.6)		22.1 (5.5)		23.7 (4.6)		20.8 (3.5)		29.3 (6.7)	
**Household income**			< 0.001		< 0.001		< 0.001		< 0.001		0.002		0.001
< 25,000	233	105.5 (25.7)		20.3 (6.3)		18.4 (7.0)		21.5 (6.1)		19.2 (4.6)		26.6 (7.7)	
25,000–69,999	260	114.1 (22.2)		21.9 (5.6)		21.5 (5.8)		22.7 (5.4)		20.3 (3.7)		27.5 (7.3)	
≥ 70,000	241	119.0 (18.6)		23.4 (4.1)		22.4 (5.1)		23.7 (4.3)		20.5 (3.7)		29.0 (6.6)	
**Insurance status**			< 0.001		< 0.001		< 0.001		< 0.001		< 0.001		0.001
Private	415	115.8 (20.6)		22.5 (5.1)		21.8 (5.6)		23.1 (4.8)		20.3 (3.7)		28.1 (7.0)	
Medicaid/Medicare	258	111.6 (23.7)		21.7(5.5)		19.8 (6.5)		22.3 (5.7)		20.0 (4.2)		27.8 (7.2)	
Uninsured	70	99.3 (28.4)		18.5 (7.1)		17.7 (7.1)		20.6 (7.1)		18.2 (5.4)		24.3 (8.5)	
Other/missing	42	113.9 (24.5)		21.2 (6.3)		21.1 (6.8)		23.4 (4.2)		19.3 (5.5)		26.8 (8.6)	
**Menopausal status**			0.001		0.01		0.22		0.72		< 0.0001		< 0.0001
Premenopausal	267	109.1 (24.3)		21.1 (6.0)		20.4 (6.6)		22.5 (5.3)		19.0 (4.6)		25.9 (7.7)	
Postmenopausal	518	114.9 (22.1)		22.2 (5.4)		20.9 (6.1)		22.7 (5.4)		20.4 (3.9)		28.5 (7.1)	
**Foreign born**			0.47		0.16		0.97		0.76		0.34		0.45
No	658	113.2 (22.8)		21.9 (5.6)		20.7 (6.2)		22.6 (5.4)		20.0 (4.2)		27.7 (7.3)	
Yes	127	111.4 (23.8)		21.2 (5.8)		20.7 (6.7)		22.8 (5.4)		19.6 (4.3)		27.1 (7.8)	
**Marital status**			0.08		0.03		0.01		0.46		0.73		0.27
Married/living as married	280	114.6 (22.6)		22.5 (5.3)		21.6 (6.0)		22.9 (5.1)		19.8 (4.1)		27.8 (7.4)	
Widow/divorced/separated	258	110.5 (23.0)		21.2 (5.8)		19.9 (6.4)		22.3 (5.7)		20.0 (4.0)		27.0 (7.4)	
Single/never married	245	113.9 (23.0)		21.9 (5.6)		20.8 (6.2)		22.7 (5.4)		20.1 (4.2)		27.9 (7.2)	
> 3 drinks/week		107.1 (21.9)		20.9 (5.1)		20.7 (5.3)		21.2 (4.9)		18.1 (5.7)		26.2 (8.3)	
**Smoking status ever**			0.17		0.36		0.04		0.36		0.004		0.95
Never	453	113.8 (22.7)		22.1 (5.6)		21.2 (6.2)		22.8 (5.4)		20.0 (4.1)		27.7 (7.4)	
Former	249	112.8 (22.8)		21.6 (5.6)		20.7 (6.0)		22.6 (5.4)		20.4 (3.9)		27.6 (7.1)	
Current (~ 24 month since diagnosis)	79	108.4 (25.3)		21.2 (5.6)		19.1 (7.1)		21.9 (5.5)		18.6 (4.9)		27.4 (7.4)	
**Alcohol 12 month since diagnosis**			0.79		0.70		0.22		0.57		0.12		0.8
Never or 1 drink/month	545	112.6 (23.0)		21.7 (5.7)		20.5 (6.4)		22.5 (5.6)		20.2 (3.9)		27.6 (7.4)	
< 3 drinks per week	190	113.5 (23.2)		22.1 (5.5)		21.2 (5.8)		23.0 (4.9)		19.5 (4.6)		27.6 (7.3)	
≥ 3 drinks per week	51	114.6 (22.3)		22.2 (5.3)		21.7 (5.8)		22.9 (4.5)		19.5 (4.9)		28.3 (7.0)	
**Physical activity (METS-hours/week) at FU1**			0.63		0.003		0.04		0.57		0.07		0.35
Q1	264	112.0 (23.5)		22.0 (5.2)		20.2 (6.7)		22.6 (5.6)		20.1 (3.9)		27.7(6.9)	
Q2	259	115.6 (21.4)		21.9 (5.5)		21.5 (5.6)		22.9 (5.2)		20.3 (3.8)		28.1 (7.5)	
Q3	262	111.4 (24.0)		21.6 (6.2)		20.7 (6.2)		22.4 (5.4)		19.5 (4.7)		27.2 (7.6)	
**Physical activity guidelines—sitting**			0.26		0.03		0.04		0.87		0.8		0.88
No	527	112.3 (22.9)		21.5 (5.8)		20.5 (6.3)		22.6 (5.4)		20.0 (4.1)		27.7 (7.2)	
Yes (high PA/low sedentary)	249	114.3 (23.3)		22.5 (5.1)		21.5 (5.9)		22.7 (5.4)		20.0 (4.4)		27.6 (7.7)	
**Hypertension**			0.38		0.01		0.12		0.91		0.59		0.98
No	273	113.9 (23.4)		22.5 (5.2)		21.2 (6.1)		22.7 (5.5)		19.8 (4.3)		27.6 (7.3)	
Yes	512	112.4 (22.9)		21.5 (5.8)		20.5 (6.3)		22.6 (5.4)		20.0 (4.1)		27.6 (7.4)	
**Diabetes**			0.03		< 0.01		< 0.01		0.07		0.66		0.55
No	570	114.0 (22.6)		22.2 (5.4)		21.2 (6.1)		22.9 (5.4)		20.0 (4.1)		27.7 (7.4)	
Yes	215	110.1 (23.8)		20.8 (6.1)		19.6 (6.5)		22.1 (5.5)		19.9 (4.4)		27.3 (7.3)	
**Lymphedema since diagnosis**			< 0.0001		< 0.0001		< 0.0001		0.97		< 0.0001		< 0.0001
No	649	115.3 (21.5)		22.4 (5.2)		21.2 (6.0)		22.7 (5.3)		20.3 (3.9)		28.6 (6.7)	
Yes	135	101.6 (26.7)		19.0 (6.8)		18.5 (6.7)		22.2 (5.9)		18.3 (5.06)		23.1 (8.4)	
**Tumor characteristics**													
**Stage AJCC**			0.08		< 0.001		0.07		0.8		0.61		0.02
0	151	114.9 (23.1)		23.0 (4.7)		21.1 (6.3)		22.5 (5.3)		20.1 (4.2)		28.2 (6.9)	
l	298	114.7 (22.2)		22.4 (5.4)		21.4 (6.1)		22.5 (5.6)		20.0 (4.3)		28.4 (7.0)	
ll	251	110.6 (23.5)		20.8 (6.1)		20.2 (6.2)		22.7 (5.5)		20.0 (4.1)		26.7 (7.8)	
lll/lV	73	109.4 (25.1)		20.7 (6.1)		19.9 (6.8)		23.1 (4.9)		19.3 (4.3)		26.4 (8.3)	
**Grade**			0.1		0.002		0.2		0.89		0.19		0.23
1	105	115.6 (20.9)		22.8 (4.7)		21.4 (6.3)		22.3 (5.9)		20.6 (3.6)		28.4 (6.7)	
2	303	114.4 (20.7)		22.4 (4.9)		21.0 (5.8)		22.8 (5.3)		20..0 (4.3)		27.9 (6.9)	
3	320	110.5 (25.1)		20.9 (6.4)		20.2 (6.7)		22.6 (5.4)		19.8 (4.3)		27.0 (7.8)	
**Tumor subtype**			0.61		< 0.01		0.97		0.95		0.82		0.72
Luminal	389	113.6 (21.6)		22.0 (5.3)		20.7 (6.0)		22.8 (5.4)		20.0 (4.1)		27.7 (7.2)	
HER2 Enriched	130	111.0 (22.4)		21.1 (5.9)		20.6 (6.1)		22.6 (5.1)		19.8 (4.5)		27.2 (7.1)	
Triple Negative	131	111.5 (27.0)		20.9 (6.8)		20.7 (6.9)		22.5 (6.0)		20.2 (4.0)		27.1 (8.5)	
Missing	135	114.3 (23.5)		23.1 (4.8)		20.9 (6.3)		22.5 (5.3)		19.8 (4.3)		28.0 (6.9)	
**Treatment information**													
**Type of surgery**			0.03		< 0.001		0.07		0.5		0.02		0.005
No surgery	24	106.2 (27.4)		18.2 (8.1)		18.7 (7.7)		22.5 (5.8)		18.9 (4.4)		27.8 (8.2)	
Lumpectomy	417	114.8 (22.5)		22.3 (5.3)		21.1 (6.0)		22.4 (5.7)		20.3 (3.9)		28.4 (7.0)	
Mastectomy	344	111.1 (23.1)		21.4 (5.7)		20.4 (6.4)		22.9 (5.04)		19.6 (4.4)		26.6 (7.6)	
**Chemotherapy**			0.01		< 0.0001		0.28		0.67		0.51		< 0.001
No	376	115.3 (21.3)		22.7 (4.9)		21.0 (6.0)		22.6 (5.3)		20.0 (4.1)		28.5 (6.8)	
Yes	409	110.8 (24.2)		21.0 (6.1)		20.5 (6.5)		22.7 (5.5)		19.9 (4.2)		26.7 (7.7)	
**Radiation therapy**			0.26		0.21		0.31		0.44		0.15		0.91
No	221	111.4 (23.9)		21.4 (6.2)		20.4 (6.6)		22.4 (5.6)		19.6 (4.5)		27.5 (7.1)	
Yes	564	113.4 (22.7)		22.0 (5.4)		20.9 (6.1)		22.7 (5.3)		20.1 (4.0)		27.6 (7.4)	
**Endocrine therapy**			0.38		0.34		0.81		0.18		0.92		0.57
No	253	111.6 (25.0)		21.5 (28.4)		20.7 (6.5)		22.3 (5.6)		19.9 (4.2)		27.4 (7.8)	
Yes	532	113.5 (22.1)		21.9 (5.3)		20.8 (6.1)		22.8 (5.3)		20.0 (4.2)		27.7 (7.1)	

*PA* physical activity

### Sociodemographic, lifestyle, and clinicopathological characteristics

Younger women with a generally lower socioeconomic status, as characterized by lower educational attainment, lack of insurance, and household income below $25,000, reported significantly lower overall QoL and domain-specific scores (*p* < 0.05). Other significant factors associated with lower overall FACT-B total scores included diabetes, presence of lymphedema, absence of surgical treatment, and receipt of chemotherapy (*p* < 0.05) (Table 1).

In subscale analyses, lower physical well-being was reported by BC survivors with hypertension, diabetes, low physical activity levels, prolonged sitting time, more advanced cancer stage at diagnosis (stages II and III/IV), and those with triple-negative BC. Lower functional well-being was significantly associated with being unmarried, current smoking, and having diabetes, lymphedema, or low physical activity levels (*p* < 0.05) (Table 1).

Additionally, lower emotional well-being was notably observed in premenopausal women, current smokers, those experiencing lymphedema, and women who did not receive surgical treatment for BC. Furthermore, lower BC subscale scores were reported by women who underwent chemotherapy and a mastectomy as part of their treatment (*p* < 0.05).

### Prospective associations of adiposity measures with overall quality of life and subscales

Table 2 shows the associations between baseline BMI, waist circumference, waist-to-hip ratio, percent body fat, and fat mass index with overall QoL and subscale scores measured at follow-up.

**Table 2:** Prospective multivariable robust Poisson regression models of adiposity measures with lower score for FACT-B and domains (reference high score) among participants in the Women’s Circle of Health Follow-Up Study (*N* = 785)

	Total	FACT-B		Physical well-being		Functional well-being		Social well-being		Emotional well-being		Breast cancer specific	
	*n* = 785												
		RR (95% CI)	P trend	RR (95% CI)	P trend	RR (95% CI)	P trend	RR (95% CI)	P trend	RR (95% CI)	P trend	RR (95% CI)	P trend
BMI (kg/m^2^)			< 0.001		< 0.001		0.01		0.05		0.40		< 0.001
Normal (18.5–24.9)	115	ref		ref		ref		ref		ref		ref	
Overweight (25.0–29.9)	212	1.15 (0.88, 1.49)		1.08 (0.84,1.37)		1.10 (0.86, 1.40)		1.01 (0.81, 1.25)		1.09 (0.86, 1.37)		1.15 (0.89, 1.49)	
Obese I (30.0–34.9)	222	1.29 (1.01, 1.65)		1.09 (0.86, 1.37)		1.08 (0.85, 1.37)		1.03 (0.83, 1.27)		1.07 (0.86, 1.34)		1.28 (1.00, 1.63)	
Obese II (35.0–39.9)	147	1.39 (1.08, 1.80)		1.22 (0.96, 1.55)		1.15 (0.90, 1.47)		1.05 (0.84, 1.31)		1.10 (0.87, 1.40)		1.47 (1.14, 1.88)	
Obese III (≥ 40.0)	89	1.54 (1.19, 2.00)		1.29 (1.00, 1.67)		1.33 (1.03, 1.70)		1.15 (0.90, 1.46)		1.20 (0.93, 1.54)		1.39 (1.06, 1.83)	
Waist circumference (cm)			0.002		< 0.001		0.03		0.20		0.69		0.003
Quartile 1 (< 90.2)	194	ref		ref		ref		ref		ref		ref	
Quartile 2 (90.2–99.4)	192	1.17 (0.94, 1.44)		0.99 (0.80, 1.23)		1.02 (0.84, 1.24)		1.03 (0.86, 1.24)		1.06 (0.87, 1.28)		1.15 (0.92, 1.43)	
Quartile 3 (99.4–108.7)	191	1.09 (0.88, 1.35)		0.96 (0.78, 1.18)		0.86 (0.69, 1.06)		0.87 (0.71, 1.06)		0.91 (0.74, 1.11)		1.18 (0.95, 1.46)	
Quartile 4 (> 108.7)	192	1.41 (1.16, 1.72)		1.26 (1.05, 1.53)		1.17 (0.98, 1.41)		1.11 (0.93, 1.32)		1.14 (0.95, 1.37)		1.44 (1.18, 1.77)	
Waist to hip ratio			0.15		0.10		0.12		0.56		0.64		0.59
Quartile 1 (0.69–0.83)	191	ref		ref		ref		red		ref		ref	
Quartile 2 (0.84–0.89)	193	1.21 (1.00, 1.48)		1.16 (0.95, 1.42)		1.06 (0.87, 1.29)		0.98 (0.81, 1.18)		1.15 (0.95, 1.39)		1.10 (0.90, 1.35)	
Quartile 3 (0.89–0.95)	194	1.06 (0.86, 1.31)		1.22 (0.99, 1.49)		1.02 (0.83, 1.24)		0.97 (0.80, 1.17)		1.08 (0.88, 1.32)		1.05 (0.85, 1.30)	
Quartile 4 (> 0.95)	191	1.19 (0.97, 1.46)		1.12 (0.91, 1.38)		1.12 (0.92, 1.36)		1.05 (0.87, 1.27)		1.22 (1.00, 1.48)		1.26 (1.03, 1.54)	
Percent body fat (%)			0.01		< 0.001		0.04		0.46		0.86		0.003
Quartile 1 (18.5–38.2)	184	ref		ref		ref		ref		ref		ref	
Quartile 2 (38.21–42.8)	185	1.03 (0.82, 1.28)		0.96 (0.77, 1.19)		0.99 (0.81, 1.22)		0.97 (0.81, 1.17)		1.05 (0.86, 1.28)		1.03 (0.82, 1.30)	
Quartile 3 (42.9–46.9)	180	1.26 (1.03, 1.55)		1.10 (0.90, 1.35)		1.05 (0.86, 1.30)		0.87 (0.71, 1.06)		1.17 (0.96, 1.42)		1.38 (1.13, 1.69)	
Quartile 4 (> 47.0)	179	1.28 (1.05, 1.57)		1.22 (1.00, 1.48)		1.15 (0.94, 1.39)		1.04 (0.87, 1.24)		1.08 (0.88, 1.31)		1.31 (1.07, 1.62)	
Fat mass index (kg/m^2^)			< 0.001		< 0.001		0.01		0.25		0.39		< 0.001
Quartile 1 (1.89–10.48)	184	ref		ref		ref		ref		ref		ref	
Quartile 2 (10.49–13.37)	181	1.04 (0.83, 1.30)		0.92 (0.73, 1.15)		1.02 (0.82, 1.26)		0.95 (0.78, 1.14)		1.01 (0.83, 1.24)		1.18 (0.94, 1.48)	
Quartile 3 (13.38–17.05)	183	1.24 (1.00, 1.53)		1.09 (0.89, 1.34)		1.13 (0.92, 1.38)		0.95 (0.79, 1.15)		1.08 (0.89, 1.31)		1.32 (1.07, 1.64)	
Quartile 4 (> 17.05)	180	1.39 (1.13, 1.70)		1.23 (1.01, 1.50)		1.21 (0.99, 1.47)		1.01 (0.84, 1.21)		1.10 (0.90, 1.34)		1.44 (1.17, 1.79)	

^a^Adjusted for age, education, marital status, menopausal status, household income, insurance, smoking, stage, subtype, surgery, and chemotherapy^b^Adiposity measures and lower score for FACT-B and domains (reference high score) at approx. 12 months after breast cancer diagnosis for participants in the Women’s Circle of Health Follow-Up Study^c^Prospective multivariable Poisson regression models with robust standard errors

Adjusted robust Poisson regression models revealed a significant positive association between higher BMI, waist circumference, percent body fat, and FMI with reduced overall QoL, as measured by the FACT-B score (*p*-value for trend < 0.05). Women with types I, II, and III obesity had an increased risk of lower QoL compared to those with normal weight, with risk ratios (RR) of 1.29 (95% CI, 1.01–1.65), 1.39 (95% CI, 1.08–1.80), and 1.54 (95% CI, 1.19–2.00), respectively. Central obesity was similarly associated with lower overall QOL. Compared to women in the lowest quartile of waist circumference, women in the highest quartile had a higher relative risk of low overall FACT-B score (RR_Q4 vs Q1_, 1.41; 95% CI, 1.16, 1.72). No statistically significant associations were observed for waist-to-hip ratio and FACT-B total score. Additionally, women in the two highest quartiles of fat mass index, Q3 (13.38–17.05 kg/m^2^) and Q4 (> 17.05 kg/m^2^), had higher risk of low total FACT-B score (RR_Q3_ 1.24, 95% CI 1.00, 1.53; and RR_Q4_ 1.39, 95% CI 1.13, 1.70), compared to women in the lowest quartile. Similar associations were observed for percent body fat and overall FACT-B score (Table 2).

### QoL subscales

Results from the adjusted robust Poisson regression revealed a significant positive trend in the association between obesity, central obesity and body composition with lower scores in physical and functional well-being, and the BC subscale score (*p*-value for trend < 0.05).

BC survivors with type III obesity at baseline showed a heightened risk of low physical and functional well-being, with RRs of 1.29 (95% CI, 1.00–1.67) and 1.33 (95% CI, 1.03–1.70), respectively. For central obesity, a marginally significant association was found with low emotional well-being among women in the highest quartile of waist-to-hip ratio (> 0.95) (RR_Q4 vs Q1_, 1.22; 95% CI, 1.00–1.48), compared to the lowest quartile, after adjusting for covariates. Compared to women in the lowest quartile of waist circumference, women in the highest quartile had a higher relative risk of low physical well-being, and BC-specific scores (RR_Q4 vs Q1_ 1.26, 95% CI 1.05, 1.53; and RR_Q4 vs Q1_ 1.44, 95% CI 1.18, 1.77, respectively). No other significant associations were observed for waist circumference and waist-to-hip ratio and QoL domains.

Regarding body composition, women in the highest quartiles of fat mass index were significantly more likely to report BC symptoms (RR_Q3 vs Q1_ 1.32, 95% CI 1.07–1.64; and RR_Q4 vs Q1_ 1.44, 95% CI 1.17–1.79) compared to women in the lowest quartile. Additionally, women in the highest quartile of fat mass index reported lower physical well-being, compared to women in the lowest quartile (RR_Q4 vs Q1_, 1.23; 95% CI, 1.01–1.50). Similar associations were observed for the associations between percent body fat and QoL domains. No other significant prospective associations were found for social, emotional, and functional well-being among Black BC survivors in our study (Table 2).

Including diabetes and hypertension in our final model partially explained the associations between type III obesity with overall FACT-B score, physical well-being, and functional well-being, but not alter the associations with BC specific symptoms. Similar results were observed for women in the highest quartile of waist circumference, waist-to-hip ratio, and fat mass index (Table 3). No differences in our results were observed after excluding women with anxiety and depression (Data not shown). STATA 18.0 was used for statistical analysis.

**Table 3:** Evaluation of the prospective associations of adiposity measures with FACT-B score and domains, further adjusting for diabetes and hypertension

	Total	FACT-B—low			Physical well-being—low			Functional well-being–low			Social Well-being—low				Emotional well-being—low				Breast cancer specific—low			
	*n* = 785																					
		RR	(95% CI)	P trend	RR	(95% CI)	P trend	RR	(95% CI)	P trend	RR	(95% CI)	P trend	RR		(95% CI)	P trend	RR		(95% CI)	P trend	
BMI (kg/m^2^)				< 0.001			< 0.0001			0.01			0.05				0.40				< 0.0001	
Normal (18.5–24.9)	115	ref			ref			ref			ref				ref				ref			
Overweight (25.0–29.9)	212	1.11 (0.85, 1.44)			1.03 (0.81, 1.31)			1.06 (0.83, 1.35)			1.00 (0.80, 1.24)				1.07 (0.85, 1.35)				1.13 (0.87, 1.47)			
Obese I (30.0–34.9)	222	1.23 (0.96, 1.58)			1.02 (0.80, 1.29)			1.02 (0.80, 1.30)			1.01 (0.82, 1.26)				1.05 (0.84, 1.32)				1.25 (0.98, 1.60)			
Obese II (35.0–39.9)	147	1.31 (1.01, 1.70)			1.12 (0.87, 1.44)			1.07 (0.83, 1.38)			1.03 (0.82, 1.30)				1.08 (0.85, 1.37)				1.43 (1.11, 1.84)			
Obese III (≥ 40.0)	89	1.44 (1.10, 1.88)			1.18 (0.91, 1.53)			1.23 (0.95, 1.58)			1.12 (0.88, 1.44)				1.17 (0.90, 1.51)				1.35 (1.03, 1.78)			
**Waist circumference (cm)**				0.002			< 0.001			0.03			0.20				0.69				< 0.001	
Quartile 1 (< 90.2)	194	ref			ref			ref			ref				ref				ref			
Quartile 2 (90.2–99.4)	192	1.14 (0.92, 1.41)			0.97 (0.78, 1.20)			1.00 (0.82, 1.22)			1.03 (0.85, 1.23)				1.05 (0.87, 1.28)				1.14 (0.92, 1.43)			
Quartile 3 (99.4–108.7)	191	1.05 (0.84, 1.30)			0.92 (0.74, 1.13)			0.82 (0.66, 1.01)			0.86 (0.70, 1.05)				0.89 (0.73, 1.10)				1.16 (0.94, 1.44)			
Quartile 4 (> 108.7)	192	1.33 (1.09, 1.63)			1.17 (0.96, 1.43)			1.10 (0.91, 1.32)			1.08 (0.90, 1.30)				1.12 (0.92, 1.35)				1.42 (1.15, 1.74)			
**Waist to hip ratio**				0.15			0.10				0.12							0.64			0.59	
Quartile 1 (0.69–0.83)	191	ref			ref			ref			ref			ref					ref			
Quartile 2 (0.84–0.89)	193	1.17 (0.96, 1.43)			1.12 (0.91, 1.37)			1.03 (0.84, 1.25)			0.97 (0.81, 1.17)			1.14 (0.94, 1.38)					1.09 (0.88, 1.34)			
Quartile 3 (0.89–0.95)	194	0.99 (0.80, 1.23)			1.13 (0.91, 1.40)			0.96 (0.78, 1.18)			0.95 (0.78, 1.16)			1.06 (0.86, 1.30)					1.02 (0.82, 1.27)			
Quartile 4 (> 0.95)	191	1.10 (0.89, 1.35)			1.02 (0.82, 1.27)			1.03 (0.84, 1.27)				1.02 (0.84, 1.25)				1.20 (0.98, 1.47)				1.22 (0.99, 1.50)		
**Percent body fat (%)**				0.01			< 0.0001				0.04			0.46				0.86				0.00
Quartile 1 (18.5–38.2)	184	ref			ref			ref				ref				ref				ref		
Quartile 2 (38.21–42.8)	185	1.00 (0.80, 1.25)			0.94 (0.75, 1.16)			0.97 (0.79, 1.19)				0.97 (0.81, 1.16)				1.04 (0.85, 1.27)				1.02 (0.81, 1.28)		
Quartile 3 (42.9–46.9)	180	1.21 (0.99, 1.49)			1.05 (0.86, 1.29)			1.02 (0.83, 1.25)				0.86 (0.70, 1.05)				1.16 (0.95, 1.40)				1.36 (1.11, 1.66)		
Quartile 4 (> 47.0)	179	1.20 (0.98, 1.47)			1.14 (0.93, 1.38)			1.08 (0.89, 1.32)				1.02 (0.85, 1.22)				1.06 (0.86, 1.29)				1.28 (1.04, 1.58)		
**Fat mass index (kg/m**^**2**^**)**				< 0.001			< 0.0001				0.01			0.25				0.39				< 0.0001
Quartile 1 (1.89–10.48)	184	ref			ref			ref				ref				ref				ref		
Quartile 2 (10.49–13.37)	181	1.00 (0.80, 1.26)			0.88 (0.71, 1.10)			0.99 (0.80, 1.22)				0.93 (0.77, 1.13)				1.00 (0.82, 1.23)				1.16 (0.92, 1.46)		
Quartile 3 (13.38–17.05)	183	1.19 (0.96, 1.47)			1.05 (0.85, 1.28)			1.09 (0.89, 1.34)				0.94 (0.78, 1.14)				1.07 (0.88, 1.30)				1.30 (1.05, 1.62)		
Quartile 4 (> 17.05)	180	1.29 (1.05, 1.59)			1.14 (0.93, 1.39)			1.13 (0.92, 1.39)				0.99 (0.82, 1.19)				1.07 (0.88, 1.32)				1.40 (1.13, 1.74)		

a Adjusted for age, education, marital status, menopausal status, household income, insurance, smoking, stage, subtype, surgery, chemotherapy, diabetes, and hypertensionb Adiposity measures and lower score for FACT-B and domains (reference high score) at approx. 12 months after breast cancer diagnosis for participants in the Women’s Circle of Health Follow-Up Studyc Prospective multivariable Poisson regression models with robust standard error

## Discussion

In this study, we evaluated prospective associations of several adiposity measures, including BMI, central obesity, and body composition with QoL in a large cohort of Black BC survivors in New Jersey. We observed that higher levels of adiposity, as measured by higher BMI, body fat, and waist circumference, were significantly associated with subsequent lower overall QoL, particularly in the physical and functional well-being domains, and the BC subscale score. The strongest associations with low QoL were observed among women with type III obesity, those in the highest quartile of waist circumference, and those with high fat mass index. Comorbidities, such as diabetes and hypertension, partially explained these associations. To our knowledge, this is one of the first studies to prospectively assess the impact of adiposity on QoL in Black women, underscoring the importance of addressing obesity as a modifiable risk factor to enhance long-term QoL in Black BC survivors.

Our study provides compelling evidence of a strong association between obesity and reduced QoL among Black BC survivors, with implications for physical and disease-specific well-being. Notably, we found that morbid obesity, central obesity, and fat distribution were significantly linked to lower overall QoL scores, diminished physical well-being, and poorer scores on BC-specific scales. Previous studies have observed that Black women report lower QoL than non-Hispanic White and Hispanic counterparts [3, 15, 27]. For instance, recent data from the Carolina Breast Cancer Study, which included 2142 women diagnosed with breast cancer from 2008 to 2013, demonstrated a lower QoL among Black women compared to White survivors [15]. Another analysis of this cohort revealed persistently lower physical and functional QoL in Black women relative to non-Hispanic White women [3]. Additionally, findings from the Health, Eating, Activity, and Lifestyle (HEAL) Study—a multi-racial and ethnic cohort of women with newly diagnosed breast cancer—indicated that African American women reported significantly lower physical functioning than both non-Hispanic White and Hispanic women, though they exhibited higher mental health scores [28]. While the direct relationship between obesity and QoL among Black BC survivors has not been extensively studied [29, 30], existing research suggests that comorbidities, particularly obesity and obesity-related conditions, correlate with poorer physical and functional QoL [31–33]. Furthermore, a possible explanation for the poor physical and BC-specific scales observed is the influence of visceral fat distribution, particularly in the central abdominal region, which has been shown to significantly influence the severity of BC symptoms after treatment [13]. This relationship potentially explains the heightened risk for a worst BC specific subscale score observed among women within the highest quartile of central obesity. Our study therefore contributes valuable insight into the compounded impact of obesity on the QoL of Black BC survivors, emphasizing the urgent need for targeted interventions and further research in this population.

Consistent with prior studies evaluating comorbidities with QoL [34–36], after adjusting for obesity-related comorbidities such as diabetes and hypertension—which are disproportionately prevalent among Black breast cancer survivors [2, 37]—we observed some attenuation in the association between adiposity QoL. However, the association persisted suggesting an independent association. These results underscore the importance of managing obesity and related comorbidities in cancer survivorship care [38]. A prior study by our team found improved comorbidity management when patients received care from shared care providers compared to the oncologist only, though only 54% of women received shared care [39]. Identifying obesity during oncologist visits may provide a valuable opportunity to improve QoL outcomes by integrating medical management, specialist referrals, and connecting patients with weight management resources, including nutritionists or survivorship programs.

Our findings support the integration of lifestyle modification strategies, particularly weight management, into survivorship care plans [40] and clinical guidelines [41, 42] to improve QoL among breast cancer survivors post-treatment. Emerging evidence underscores the role of structured exercise and dietary interventions in mitigating the adverse effects of obesity and excess body fat, ultimately enhancing QoL [34, 43–46] and often face structural and contextual barriers to healthy behaviors [47, 48]. Targeted interventions addressing these challenges have the potential to improve physical well-being and overall QoL during survivorship [44, 45, 49].

Additionally, it is vital to address the unique challenges and barriers to adherence to lifestyle modification programs, particularly among Black BC survivors with obesity, who often face multiple comorbidities [44] and are underrepresented in research.

This study has several strengths, including the examination of the prospective association between obesity, fat distribution, and QoL in a population-based cohort of Black BC survivors—a group frequently underrepresented in research. While a few other studies have explored the relationship between excess body weight and QoL in this population [29, 34, 50], few have done so prospectively [51], which enhances the internal validity of our findings by reducing the risk of reverse causation. However, some limitations warrant consideration. Although our models were adjusted for key clinical and treatment-related factors, residual confounding due to unmeasured variables may have influenced the observed associations. Despite these limitations, our study provides valuable insights into the influence of obesity on QoL among Black BC survivors. Future research should focus on elucidating the mechanisms underlying these associations and identifying effective strategies to mitigate the adverse effects of obesity on survivorship outcomes.

## Conclusion

We observed a significant prospective association of adiposity with QoL in Black BC survivors. Morbid obesity and central fat distribution are strongly associated with reduced overall QoL, compromised physical well-being, and exacerbated BC symptoms. Addressing adiposity through tailored interventions that consider the unique survivorship, physical, and psychosocial factors affecting QoL in Black women is crucial for improving BC outcomes. In conclusion, this study underscores the critical need to maintain a healthy weight to enhance and sustain physical well-being and overall QoL for Black women during BC survivorship.

## Data Availability

No datasets were generated or analysed during the current study.
